# Aclidinium bromide and formoterol fumarate as a fixed-dose combination in COPD: pooled analysis of symptoms and exacerbations from two six-month, multicentre, randomised studies (ACLIFORM and AUGMENT)

**DOI:** 10.1186/s12931-015-0250-2

**Published:** 2015-08-02

**Authors:** Eric D. Bateman, Kenneth R. Chapman, Dave Singh, Anthony D. D’Urzo, Eduard Molins, Anne Leselbaum, Esther Garcia Gil

**Affiliations:** Division of Pulmonology, Department of Medicine, University of Cape Town, George Street, Mowbray, 7700 Cape Town South Africa; Asthma & Airway Centre, University Health Network, Toronto Western Hospital, Toronto, ON Canada; University of Manchester, Medicines Evaluation Unit, University Hospital of South Manchester, Manchester, UK; Department of Family and Community Medicine, University of Toronto, Toronto, ON Canada; R&D Centre, AstraZeneca PLC (former employee of Almirall S.A.), Barcelona, Spain; Former employee of Almirall S.A., Barcelona, Spain

**Keywords:** Aclidinium bromide/formoterol fumarate, Chronic obstructive pulmonary disease, Fixed-dose combination, Symptoms

## Abstract

**Background:**

The combination of aclidinium bromide, a long-acting anticholinergic, and formoterol fumarate, a long-acting beta_2_-agonist (400/12 μg twice daily) achieves improvements in lung function greater than either monotherapy in patients with chronic obstructive pulmonary disease (COPD), and is approved in the European Union as a maintenance treatment. The effect of this combination on symptoms of COPD and exacerbations is less well established. We examined these outcomes in a pre-specified analysis of pooled data from two 24-week, double-blind, parallel-group, active- and placebo-controlled, multicentre, randomised Phase III studies (ACLIFORM and AUGMENT).

**Methods:**

Patients ≥40 years with moderate to severe COPD (post-bronchodilator forced expiratory volume in 1 s [FEV_1_]/forced vital capacity <70 % and FEV_1_ ≥30 % but <80 % predicted normal) were randomised (ACLIFORM: 2:2:2:2:1; AUGMENT: 1:1:1:1:1) to twice-daily aclidinium/formoterol 400/12 μg or 400/6 μg, aclidinium 400 μg, formoterol 12 μg or placebo via Genuair™/Pressair®. Dyspnoea (Transition Dyspnoea Index; TDI), daily symptoms (EXAcerbations of Chronic pulmonary disease Tool [EXACT]-Respiratory Symptoms [E-RS] questionnaire), night-time and early-morning symptoms, exacerbations (Healthcare Resource Utilisation [HCRU] and EXACT definitions) and relief-medication use were assessed.

**Results:**

The pooled intent-to-treat population included 3394 patients. Aclidinium/formoterol 400/12 μg significantly improved TDI focal score versus placebo and both monotherapies at Week 24 (all *p* < 0.05). Over 24 weeks, significant improvements in E-RS total score, overall night-time and early-morning symptom severity and limitation of early-morning activities were observed with aclidinium/formoterol 400/12 μg versus placebo and both monotherapies (all *p* < 0.05). The rate of moderate or severe HCRU exacerbations was significantly reduced with aclidinium/formoterol 400/12 μg compared with placebo (*p* < 0.05) but not monotherapies; the rate of EXACT-defined exacerbations was significantly reduced with aclidinium/formoterol 400/12 μg versus placebo (*p* < 0.01) and aclidinium (*p* < 0.05). Time to first HCRU or EXACT exacerbation was longer with aclidinium/formoterol 400/12 μg compared with placebo (all *p* < 0.05) but not the monotherapies. Relief-medication use was reduced with aclidinium/formoterol 400/12 μg versus placebo and aclidinium (*p* < 0.01).

**Conclusions:**

Aclidinium/formoterol 400/12 μg significantly improves 24-hour symptom control compared with placebo, aclidinium and formoterol in patients with moderate to severe COPD. Furthermore, aclidinium/formoterol 400/12 μg reduces the frequency of exacerbations compared with placebo.

**Trial registration:**

NCT01462942 and NCT01437397 (ClinicalTrials.gov)

**Electronic supplementary material:**

The online version of this article (doi:10.1186/s12931-015-0250-2) contains supplementary material, which is available to authorized users.

## Background

Patients with chronic obstructive pulmonary disease (COPD) experience a range of troublesome symptoms, including breathlessness (dyspnoea), cough and chest tightness [1–3] and from time to time episodes of acute worsening of symptoms, requiring a change in medication and even hospitalisation (exacerbations) [4]. These exacerbations are associated with accelerated decline in lung function [5–7], reduced health status (quality of life) [8–10] and higher mortality rates [4, 7].

The symptoms of COPD are most severe during the night and early morning [2]. Observational studies suggest that approximately 40–81 % of patients experience morning symptoms [11, 12] and 58–78 % of patients experience night-time disturbances or symptoms [3, 11, 12]. There are several potential consequences of these symptoms. Morning symptoms can be associated with limitation of activities during the day, impaired health status and increased risk of an exacerbation [12]. Night-time symptoms disturb sleep and reduce sleep quality, and, in the long term, may be associated with development or worsening of cardiovascular disease, cognitive problems, depression and increased mortality [1]. Reducing the prevalence and severity of COPD symptoms during the day and night, including the early morning, is an important goal of treatment.

The use of one or two long-acting bronchodilators is recommended for patients in Global initiative for chronic Obstructive Lung Disease (GOLD) groups B, C and D [4], who are symptomatic and/or at higher risk of COPD exacerbations based on their history over the previous year. Compared with single bronchodilators, combining bronchodilators with complementary mechanisms of action, such as a long-acting muscarinic antagonist (LAMA) and a long-acting ß_2_-agonist (LABA), achieves greater improvements in lung function than either bronchodilator given alone. Combining two bronchodilators in one device has the potential to improve patient adherence to treatment [13, 14] and may result in greater symptom control. However, results of studies of symptom relief and prevention of exacerbations with dual bronchodilators compared with single bronchodilator use or placebo have varied [15–19]. Effects on patient-reported outcomes (PROs) have generally been lower than anticipated and this may be due to a number of factors. PROs often exhibit a large placebo response, which can make interpretation of treatment effect difficult [20, 21]. In addition, it may be difficult to observe significant treatment effects with a dual bronchodilator versus its monotherapy components, given that the added benefit of a second bronchodilator is likely to be much lower than that seen with a single bronchodilator versus placebo [21].

A fixed-dose combination (FDC) of the LAMA, aclidinium and the LABA, formoterol fumarate, 400/12 μg twice daily (BID) has recently been approved in the European Union as a maintenance bronchodilator for patients with COPD [22]. Two pivotal, randomised, placebo-controlled studies (ACLIFORM and AUGMENT) of two different doses of aclidinium/formoterol FDC (400/12 μg and 400/6 μg BID) have previously been reported [23, 24]. In these studies, rapid and sustained improvements in lung function were observed over 24 h, with significant improvements in bronchodilation compared with placebo and the monotherapies across six months. In both studies, the safety profiles of the aclidinium/formoterol FDCs were similar to placebo and the monotherapies, with no evidence for additive adverse events (AEs) [23, 24]. The co-primary endpoints of both studies were 1-hour morning post-dose forced expiratory volume in 1 s (FEV_1_) versus aclidinium and morning pre-dose (trough) FEV_1_ versus formoterol. These and other pre-specified secondary and additional endpoints, including patient-reported outcomes, have been reported elsewhere [23, 24].

Here, we report results of a pre-specified pooled analysis of data from the two studies, which together are powered to provide more reliable estimates of the effect of aclidinium/formoterol FDC on symptoms and COPD exacerbations compared not only with placebo, but also the monotherapies, across a wide range of endpoints. We focus on data from patients using the approved 400/12 μg dose.

## Methods

### Study design

ACLIFORM and AUGMENT were Phase III, double-blind, randomised, parallel-group, active- and placebo-controlled multicentre studies, conducted at 193 centres in 22 countries (South Africa, South Korea and 20 countries in Europe) and 222 centres in 4 countries (Australia, Canada, New Zealand and the USA), respectively. Each study had a 2- to 3-week run-in period (during which long-acting bronchodilator medications were withdrawn) followed by a 24-week treatment period and a follow-up visit two weeks after treatment concluded. Patients were randomised in a 2:2:2:2:1 ratio in ACLIFORM and a 1:1:1:1:1 ratio in AUGMENT to receive aclidinium/formoterol FDC 400/12 μg or 400/6 μg, aclidinium 400 μg, formoterol 12 μg or placebo (all BID) via a multidose dry powder inhaler (Genuair™/Pressair®^1^; AstraZeneca PLC, Barcelona, Spain).

The studies were conducted in accordance with the Declaration of Helsinki, International Conference on Harmonisation/Good Clinical Practice Guidelines and local regulations. The protocols were approved by the regulatory authorities in each country and the Institutional Review Board or Independent Ethics Committee at each study centre.

### Study populations

Patients with stable COPD aged ≥40 years who were current or former cigarette smokers (smoking history ≥10 pack-years) and diagnosed with moderate to severe airflow obstruction (post-bronchodilator FEV_1_/forced vital capacity <70 % and FEV_1_ ≥ 30 % and <80 % predicted) were eligible for inclusion. Key exclusion criteria included COPD exacerbation or respiratory tract infection ≤6 weeks pre-screening (≤3 months if hospitalised), presence of clinically significant respiratory disease other than COPD or clinically significant cardiovascular conditions (defined as myocardial infarction ≤6 months pre-screening, unstable angina or unstable arrhythmia which required changes in therapy within 12 months pre-screening or newly-diagnosed arrhythmia ≤3 months pre-screening, hospitalisation ≤12 months pre-screening for heart failure functional class III or IV as per the New York Heart Association guidelines). A COPD exacerbation in the previous 12 months was not a requirement for inclusion.

Inhaled salbutamol was permitted as relief medication; its use was discontinued 6 h prior to study visits. Additional permitted medications included inhaled corticosteroids (ICS), oral or parenteral corticosteroids (≤10 mg/day of prednisone or 20 mg every other day), oral sustained-release theophylline and oxygen therapy (<15 h/day), provided treatment was stable ≥4 weeks pre-screening.

### Study assessments and endpoints

Dyspnoea was assessed using the Baseline Dyspnoea Index; changes were measured using the Transitional Dyspnoea Index (TDI). TDI endpoints included TDI focal score and a responder analysis quantifying responders and ‘deteriorators’ (percentage of patients with improvements or worsenings of ≥1 unit [the minimum clinically important difference, MCID]) at Weeks 4, 12 and 24.

Symptoms and relief-medication use were recorded in an electronic patient diary. Daily COPD symptoms were assessed using the EXAcerbations of Chronic pulmonary disease Tool (EXACT)-Respiratory Symptoms (E-RS) questionnaire (11 items from the 3 domains of the 14-item EXACT questionnaire: breathlessness, cough and sputum and chest symptoms) completed at night [25, 26]. E-RS total scores range from 0 to 40, with higher scores indicating more severe symptoms. Changes from baseline in E-RS total score and breathlessness, cough and sputum and chest symptoms domain scores were assessed over 24 weeks. E-RS responders were also analysed using the definition recently proposed by Leidy and colleagues (percentage of patients achieving a reduction in E-RS total score of ≥2 units) [27]. Night-time and early-morning symptoms were recorded every morning using newly developed questionnaires. The psychometric properties of these questionnaires have been evaluated and final tools developed (the Early-Morning Symptoms of COPD Instrument [EMSCI] and the Night-time Symptoms of COPD Instrument [NiSCI]) [28, 29]. Scores ranged from 0 (no symptoms) to 4 (very severe symptoms). The questionnaires also evaluated nocturnal awakenings and limitation of early-morning activities (scores ranged from 0 [no limitation] to 4 [a very great deal]). Symptoms assessed over 24 weeks included change from baseline in the severity of night-time and early-morning cough, wheezing, shortness of breath and difficulty bringing up phlegm, overall night-time and early-morning symptom severity, number of nocturnal awakenings and limitation of early-morning activities due to COPD symptoms. Change from baseline in daily relief-medication use over 24 weeks was also assessed using the patient diary.

COPD exacerbations were assessed throughout the study by the Healthcare Resource Utilisation (HCRU) definition and the 14-item EXACT questionnaire [25]. An HCRU exacerbation was defined as an increase in COPD symptoms during ≥2 consecutive days that required a change in COPD treatment, and an EXACT exacerbation was defined as a persistent increase from baseline in total EXACT score of ≥9 points for ≥3 days or ≥12 points for ≥2 days. HCRU exacerbations were categorised as mild (self-managed by the patient at home by increasing usual COPD medication [short-acting bronchodilator and/or ICS use]), moderate (not leading to hospitalisation but treated with antibiotics and/or systemic corticosteroids [or increase in dose of systemic corticosteroids]) or severe (leading to hospitalisation [overnight stay or emergency room visit]). Exacerbation endpoints were the rate of COPD exacerbation and time to first COPD exacerbation.

TDI focal score versus placebo at Week 24 was a secondary endpoint in both studies; all other assessments listed above were additional endpoints.

### Statistical analyses

Data were analysed using SAS® Version 9.3. All efficacy analyses were pre-specified, other than those of TDI deteriorators, E-RS responders and data stratified by GOLD group or concomitant ICS use. All analyses were performed in the intent-to-treat (ITT) population (patients who had a baseline and ≥1 post-baseline FEV_1_ measurement and received ≥1 dose of the study medication), with the exception of exacerbations analyses, which were performed in the ITT-exacerbations population (that is, all randomised patients who received ≥1 dose of study medication).

TDI focal score and three dimension scores, E-RS total score and domain scores, night-time and early-morning symptom severity and relief-medication use were analysed by a mixed model for repeated measures, and TDI responders/deteriorators and E-RS responders were analysed by a logistic random-effect model. These models were adjusted by age and baseline values as covariates, and treatment group, sex, smoking status, visit and treatment group-by-visit interaction as fixed-effect factors. Additionally, the logistic random-effect model had a random intercept to account for the variability amongst patients. The rate of COPD exacerbations per patient per year and time to first exacerbation were analysed by a negative binomial regression model and a Cox proportional hazard model, respectively. The models included age as a covariate, and treatment group, sex, baseline ICS use, baseline COPD severity and smoking status as factors. In addition, TDI and exacerbations data were stratified by concomitant ICS use (defined as any ICS use at baseline [in the 15 days prior to study start] that continued throughout the treatment period) and GOLD group (based on airflow limitation, exacerbation risk and SGRQ total score [a surrogate measure for the COPD Assessment Test (CAT); an SGRQ total score ≥25 corresponds with a CAT score ≥10 [30]]).

## Results

### Patient population

**Fig. 1:**
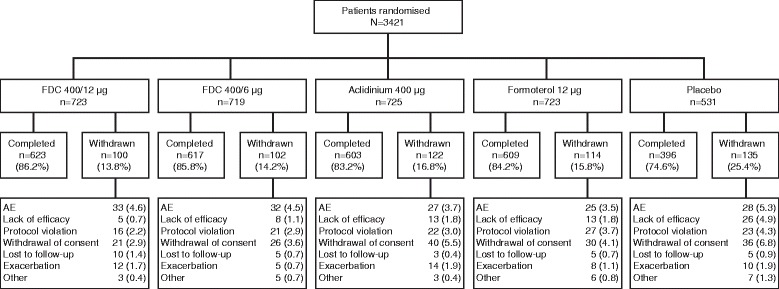
CONSORT diagram of patient flow in the pooled ACLIFORM and AUGMENT studies. Data for the 400/6 μg dose of the FDC are not reported in this paper and can be found elsewhere [23, 24]; AE, adverse event; FDC, aclidinium/formoterol fixed-dose combination

Of 3421 randomised patients, 3394 (99.2 %) were included in the ITT population and 3398 (99.3 %) were included in the ITT-exacerbations population. In total, 573 (16.7 %) patients discontinued treatment; the primary reasons were withdrawal of consent (4.5 %), AEs (4.2 %) and protocol violation (3.2 %). Patient flow and the reasons for discontinuation are presented by treatment group in Fig. 1. Patient demographics and baseline characteristics were similar between the treatment groups (Table 1). The patient population was not enriched for exacerbations and the number of exacerbations in the previous year was low (0.3–0.5 exacerbations; Table 1). Overall, 1335 (39.3 %) patients in the ITT-exacerbations population were using ICS at baseline. At baseline, 88.3 % and 94.4 % of patients reported night-time and early-morning symptoms of COPD (Table 2), although symptoms were relatively mild (rated 1.1–1.3 units out of a maximum score of 4; Table 1). When the ITT population was stratified by GOLD group, 9.2, 45.9, 3.0 and 41.9 % of patients were in GOLD groups A, B, C and D, respectively (75 patients were missing data required for GOLD classification). Baseline GOLD data by treatment group are shown in Table 1.

**Table 1:** Patient demographics and baseline characteristics for the pooled ACLIFORM and AUGMENT studies

	FDC 400/12 μg (*n* = 720)	Aclidinium 400 μg (*n* = 720)	Formoterol 12 μg (*n* = 715)	Placebo (*n* = 525)
Age, years	63.4 ± 8.5	63.7 ± 8.5	63.5 ± 8.2	63.7 ± 8.6
Gender, male, n (%)	429 (59.6)	442 (61.4)	423 (59.2)	313 (59.6)
Current smoker, n (%)	354 (49.2)	351 (48.8)	350 (49.0)	263 (50.1)
FEV_1_, L	1.4 ± 0.5	1.4 ± 0.5	1.4 ± 0.5	1.4 ± 0.6
Post-bronchodilator FEV_1_, % predicted	53.9 ± 13.2	53.3 ± 13.1	54.2 ± 13.1	53.5 ± 13.4
Number of exacerbations in previous 12 months	0.5 (0.9)	0.5 (0.8)	0.4 (0.8)	0.3 (0.7)
Prior COPD medication^a^, n (%)				
Any COPD medication	576 (80.0)	591 (82.1)	574 (80.3)	411 (78.3)
LABA + ICS	210 (29.2)	234 (32.5)	222 (31.0)	172 (32.8)
LAMA	209 (29.0)	190 (26.4)	181 (25.3)	141 (26.9)
ICS	114 (15.8)	106 (14.7)	96 (13.4)	62 (11.8)
LABA	85 (11.8)	81 (11.3)	92 (12.9)	42 (8.0)
BDI focal score	6.4 ± 2.1	6.5 ± 2.1	6.4 ± 2.2	6.5 ± 2.2
E-RS total score^b^	12.9 ± 6.8	12.5 ± 6.4	12.3 ± 6.6	12.2 ± 6.3
Overall night-time COPD symptom severity score^c^	1.1 ± 0.7	1.1 ± 0.7	1.1 ± 0.7	1.1 ± 0.7
Overall early-morning COPD symptom severity score^c^	1.3 ± 0.7	1.3 ± 0.7	1.2 ± 0.7	1.2 ± 0.6
GOLD group, n (%)				
A	62 (8.8)	51 (7.3)	74 (10.6)	48 (9.4)
B	320 (45.5)	322 (46.0)	327 (46.7)	224 (43.7)
C	16 (2.3)	27 (3.9)	16 (2.3)	17 (3.3)
D	305 (43.4)	300 (42.9)	283 (40.4)	224 (43.7)

Data are presented as mean ± standard deviation for the pooled ITT population, unless otherwise stated

*BDI* Baseline Dyspnoea Index, *COPD* chronic obstructive pulmonary disease, *E-RS* EXAcerbations of Chronic obstructive pulmonary disease Tool (EXACT)-Respiratory Symptoms questionnaire, *FDC* aclidinium/formoterol fixed-dose combination, *FEV*_*1*_ forced expiratory volume in 1 s, *ICS* inhaled corticosteroid, *ITT* intent-to-treat, *LABA* long-acting β_2_-agonist, *LAMA* long-acting muscarinic antagonist

^a^Patients can be included in multiple categories

^b^E-RS total scores range from 0 to 40 with higher scores indicating more severe symptoms

^c^Night-time and early-morning symptom scores range from 0 (no symptoms) to 4 (very severe symptoms)

**Table 2:** Prevalence of daytime, night-time and early-morning symptoms at baseline

	Patients with symptoms (%)
Night-time symptoms	
Any	88.3
Cough	72.7
Wheezing	59.3
Shortness of breath	67.2
Difficulty bringing up phlegm	44.0
Nocturnal awakenings	57.4
Early-morning symptoms	
Any	94.4
Cough	81.5
Wheezing	57.4
Shortness of breath	77.6
Difficulty bringing up phlegm	48.6
Limitation of early-morning activities	90.6

Data are for the pooled ITT population

*ITT* intent-to-treat

### Dyspnoea (TDI)

**Fig. 2:**
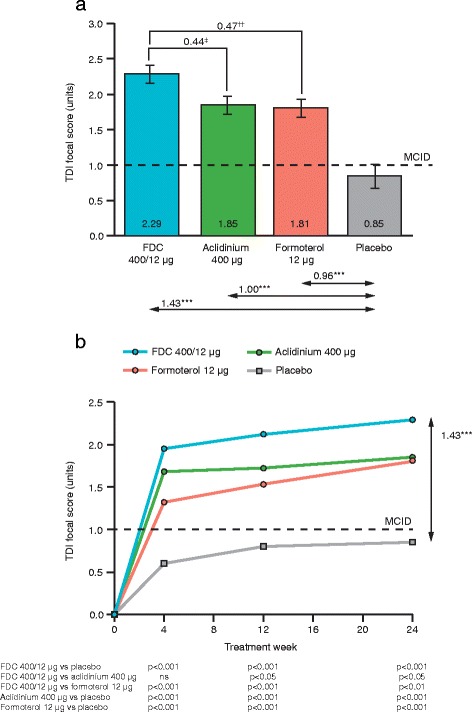
TDI focal score at Week 24 (**a**) and over 24 weeks (**b**). Data are LS means ± SE for the pooled ITT population; ^***^ *p* < 0.001 vs placebo, ^‡^ *p* < 0.05 vs aclidinium ^††^ *p* < 0.01 vs formoterol; FDC, aclidinium/formoterol fixed-dose combination; ITT, intent-to-treat; LS, least squares; MCID, minimum clinically important difference; ns, not significant; SE, standard error; TDI, Transitional Dyspnoea Index

FDC 400/12 μg significantly improved TDI focal score versus placebo at all visits assessed, and these improvements exceeded the MCID of 1 unit (range of least squares [LS] means differences vs placebo: 1.32–1.43 units; all time points *p* < 0.001). Additionally, FDC 400/12 μg significantly improved TDI focal score versus formoterol at all visits assessed (range of LS means differences vs formoterol: 0.47–0.63 units; all visits *p* < 0.01) and versus aclidinium from Week 12 onwards (difference vs aclidinium: 0.39–0.44 units; Week 12 and 24 *p* < 0.05; Fig. 2a and b).

Overall, 61.9 % of patients achieved the MCID in TDI focal score with FDC 400/12 μg compared with 55.7 % for aclidinium, 57.0 % for formoterol and 40.3 % for placebo. Treatment with FDC 400/12 μg significantly increased the odds of improving by the MCID in TDI versus placebo (Table 3), and significantly reduced the odds of TDI worsening by the MCID versus placebo (Table 3). There were no significant differences in the odds of TDI improving or worsening by the MCID with FDC 400/12 μg versus either monotherapy.

**Table 3:** TDI responders/deteriorators at Week 24

	FDC 400/12 μg (*n* = 607)	Aclidinium 400 μg (*n* = 596)	Formoterol 12 μg (*n* = 596)	Placebo (*n* = 384)
Patients with ≥1 unit improvement in TDI, %	61.9	55.7	57.0	40.3
OR vs placebo	2.8^***^	2.1^***^	2.2^***^	-
OR vs aclidinium	1.3	-	-	-
OR vs formoterol	1.3	-	-	-
Patients with ≤1 unit worsening in TDI, %	7.8	9.3	10.9	15.8
OR vs placebo	0.4^***^	0.6^**^	0.7	-
OR vs aclidinium	0.8	-	-	-
OR vs formoterol	0.7	-	-	-

Data are for the pooled ITT population; MCID for TDI is ≥1 unit

*COPD* chronic obstructive pulmonary disease, *FDC* aclidinium/formoterol fixed-dose combination, *ITT* intent-to-treat, *OR* odds ratio, *TDI* Transition Dyspnoea Index

^***^ *p* < 0.001, ^**^ *p* < 0.01 vs placebo

When stratified by ICS use, FDC 400/12 μg significantly improved TDI versus placebo regardless of concomitant ICS use (LS mean difference vs placebo with ICS: 1.59 units, *p* < 0.001; without ICS: 1.36 units, *p* < 0.001). Significant improvements in TDI with FDC 400/12 μg versus the monotherapies were only observed in patients who were not using concomitant ICS (LS mean difference vs aclidinium with ICS: 0.02 units, *p* = 0.948; without ICS: 0.66, *p* = 0.002; LS mean difference vs formoterol with ICS: 0.45 units, *p* = 0.105; without ICS: 0.48, *p* = 0.024).

Data for improvements in TDI at Week 24 stratified by GOLD group are presented in Additional file 1.

### Daily symptoms (E-RS)

Over 24 weeks, E-RS total score was significantly improved with FDC 400/12 μg compared with placebo and both monotherapies (mean change from baseline: FDC 400/12 μg: −2.4 units [−18.6 %]; aclidinium: −1.8 units [−14.2 %]; formoterol: −1.8 units [−14.7 %]; placebo: –1.2 units [−10.0 %]; *p* < 0.001 vs placebo; *p* < 0.01 vs both monotherapies; Additional file 1: Figure S2). Overall, 48.7 % of patients had improvements in E-RS total score that exceeded the recently proposed MCID (percentage of patients achieving a reduction in E-RS total score of ≥2 units) [27] compared with 41.3 % with aclidinium, 42.3 % with formoterol and 34.4 % with placebo. Treatment with FDC 400/12 μg significantly increased the odds of improving by the MCID versus placebo (odds ratio [OR]: 1.9; *p* < 0.001) and formoterol (OR: 1.3; *p* < 0.05) but not aclidinium (OR: 1.2; *p* = 0.145). Improvements in E-RS domain scores are described in an online supplement (Additional file 1).

### Night-time and early-morning symptoms (NiSCI and EMSCI)

**Fig. 3:**
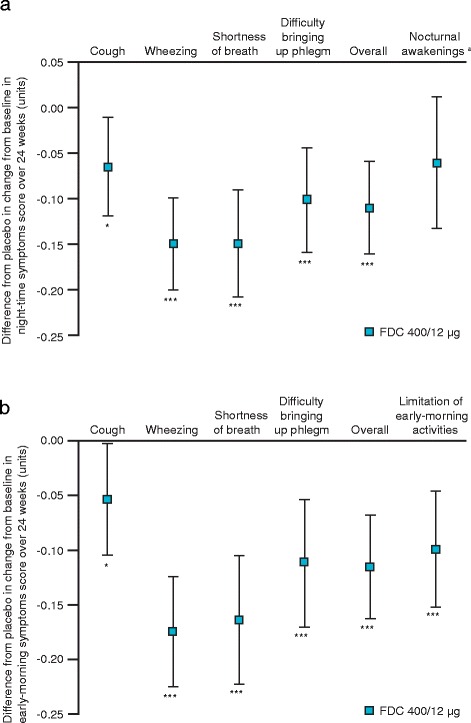
Difference from placebo in change from baseline in symptom severity over 24 weeks. **a** Night-time symptoms; **b** early-morning symptoms; Data are LS means differences from placebo ± 95 % CIs for the pooled ITT population; ^*^ *p* < 0.05, ^***^ *p* < 0.001 vs placebo; ^a^Nocturnal awakenings were the average number of awakenings per night. Other night-time symptoms were measured on a scale from 0 (no symptoms) to 4 (very severe symptoms). Larger negative values indicate greater improvements in symptom severity; CI, confidence interval; FDC, aclidinium/formoterol fixed-dose combination; ITT, intent-to-treat; LS, least squares

Over 24 weeks, FDC 400/12 μg significantly improved night-time and early-morning symptom severity compared with placebo, including both overall and individual symptom severity scores (cough, wheezing, shortness of breath and difficulty bringing up phlegm; Fig. 3a and b). Additionally, compared with placebo, FDC 400/12 μg significantly improved limitation of activities due to morning symptoms (Fig. 3b). FDC 400/12 μg had no significant effect on nocturnal awakenings compared with placebo (Fig. 3a).

Although improvements in overall night-time symptom severity were observed in all treatment arms, the change from baseline was significantly greater with FDC 400/12 μg compared with the monotherapies (FDC 400/12 μg: −0.25 units [−21.6 %]; aclidinium 400 μg: –0.16 units [−14.5 %]; formoterol 12 μg: −0.19 units [−18.2 %]; *p* < 0.001 vs aclidinium and *p* < 0.05 vs formoterol). A similar pattern was observed for improvements from baseline in overall early-morning symptom severity (FDC 400/12 μg: −0.23 units [−17.0 %]; aclidinium 400 μg: −0.14 units [−10.7 %]; formoterol 12 μg: −0.17 units [−13.6 %]; *p* < 0.001 vs aclidinium and *p* < 0.01 vs formoterol).

Changes in individual night-time and early-morning symptoms (cough, wheezing, shortness of breath and difficulty bringing up phlegm), limitation of early-morning activities and nocturnal awakenings versus monotherapy are described in an online supplement (Additional file 1).

### COPD exacerbations

**Fig. 4:**
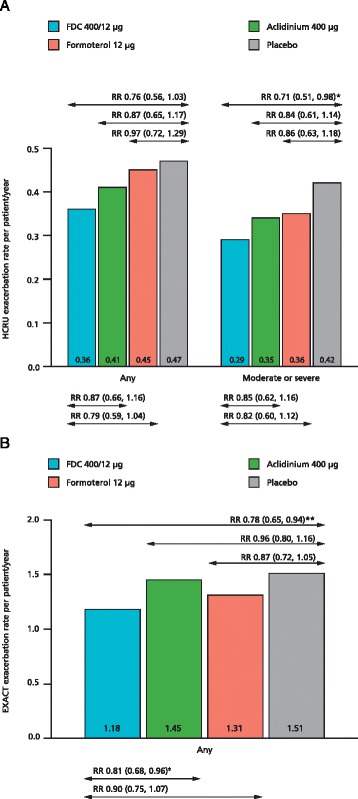
Rate of COPD exacerbations based on HCRU (**a**) and EXACT (**b**) definitions. Data are LS means and RR (CI) for the pooled ITT-exacerbations population; ^*^ *p* < 0.05, ^**^ *p* < 0.01 vs placebo, ^ǂ^ *p* < 0.05 vs aclidinium; CI, confidence interval; COPD, chronic obstructive pulmonary disease; EXACT; EXAcerbations of Chronic pulmonary disease Tool; FDC, aclidinium/formoterol fixed-dose combination; HCRU, Healthcare Resource Utilisation; ITT, intent-to-treat; LS, least squares; RR, rate ratio

The rate of moderate or severe HCRU exacerbations was significantly lower (−29 %) with FDC 400/12 μg compared with placebo. The rate of HCRU exacerbations of any severity was also lower (−24 %) with FDC 400/12 μg compared with placebo; however, the differences did not reach statistical significance (Fig. 4a). Additionally, compared with placebo, FDC 400/12 μg significantly increased the time to first exacerbation for HCRU exacerbations of any severity, and also those that were moderate or severe (Table 4). These results were supported by the EXACT data, where higher rates of exacerbations were observed (1.18–1.51 EXACT exacerbations [any severity] compared with 0.36–0.47 HCRU exacerbations [any severity] per patient per year across treatment groups) and a similar pattern of reduction in exacerbation rate (−22 %) and time to first exacerbation was seen with FDC 400/12 μg versus placebo (Fig. 4b; Table 4). Monotherapy data for rate of exacerbation and time to first exacerbation are also shown in Fig. 4 and Table 4, respectively.

**Table 4:** Time to first COPD exacerbation based on HCRU and EXACT

	FDC 400/12 μg (*n* = 720)	Aclidinium 400 μg (*n* = 722)	Formoterol 12 μg (*n* = 716)
Time to first HCRU exacerbation of any severity			
HR vs placebo (95 % CI)	0.72 (0.53, 0.97)^*^	0.86 (0.64, 1.14)	0.94 (0.71, 1.25)
HR vs aclidinium (95 % CI)	0.84 (0.63, 1.12)	-	-
HR vs formoterol (95 % CI)	0.77 (0.58, 1.01)	-	-
Time to first HCRU exacerbation of moderate to severe severity			
HR vs placebo (95 % CI)	0.70 (0.51, 0.96)^*^	0.84 (0.62, 1.14)	0.90 (0.67, 1.22)
HR vs aclidinium (95 % CI)	0.83 (0.61, 1.13)	-	-
HR vs formoterol (95 % CI)	0.77 (0.57, 1.05)	-	-
Time to first EXACT exacerbation of any severity			
HR vs placebo (95 % CI)	0.79 (0.65, 0.95)^*^	0.92 (0.76, 1.10)	0.85 (0.71, 1.03)
HR vs aclidinium (95 % CI)	0.86 (0.72, 1.03)	-	-
HR vs formoterol (95 % CI)	0.92 (0.77, 1.10)	-	-

Data are for the pooled ITT-exacerbations population

*CI* confidence interval, *COPD* chronic obstructive pulmonary disease, *EXACT* EXAcerbations of Chronic obstructive pulmonary disease Tool, *FDC* aclidinium/formoterol fixed-dose combination, *HCRU* Healthcare Resource Utilisation, *HR* hazard ratio, *ITT* intent-to-treat

^*^ *p* < 0.05 vs placebo

Data for rate of exacerbations stratified by ICS use are presented in Additional file 1. When HCRU or EXACT exacerbations were stratified by concomitant ICS use, patients with concomitant ICS use had higher exacerbation rates compared with those who were not using ICS (Additional file 1: Figure S4). The reduction in rate of moderate to severe HCRU exacerbations and HCRU exacerbations of any severity was significantly greater with FDC 400/12 μg compared with placebo in patients with concomitant ICS use but not those without ICS; the comparisons versus the monotherapies did not reach significance in patients with or without concomitant ICS use (Additional file 1: Figure S4). Similar results were obtained for EXACT exacerbations, although in addition, FDC 400/12 μg significantly reduced the rate of EXACT exacerbations compared with aclidinium 400 μg (Additional file 1: Figure S4).

Data for exacerbation rates stratified by GOLD group are presented in Additional file 1.

### Relief-medication use

Treatment with FDC 400/12 μg reduced overall daily relief-medication use compared with placebo and the monotherapies, although only the comparisons versus placebo and aclidinium reached statistical significance (mean [CI]: FDC 400/12 μg: −1.73 [−1.88, −1.57] puffs/day; aclidinium 400 μg: −1.37 [−1.52, −1.21] puffs/day; formoterol 12 μg: −1.52 [−1.68, −1.37] puffs/day; placebo: −0.82 [−1.00, −0.63] puffs/day; *p* < 0.001 vs placebo, *p* < 0.01 vs aclidinium).

## Discussion

This pooled analysis of the ACLIFORM and AUGMENT studies showed that over six months, treatment with aclidinium/formoterol FDC 400/12 μg led to reduced breathlessness compared with placebo and both monotherapies in patients with moderate to severe COPD. Improvements in daily symptoms (E-RS total score) and night-time and early-morning symptoms (overall NiSCI and EMSCI symptom scores) were also observed versus placebo and monotherapies. The FDC also significantly reduced relief-medication use versus placebo and aclidinium. The rate of exacerbations (moderate or severe HCRU exacerbations and EXACT exacerbations of any severity) was also significantly reduced compared with placebo, but not the monotherapies, with the exception of the rate of EXACT exacerbations, which was significantly reduced with FDC 400/12 μg compared with aclidinium 400 μg.

These findings help to establish the clinical relevance of the lung function improvements previously reported from the ACLIFORM and AUGMENT studies, where clinically and statistically significant improvements in bronchodilation were observed with both aclidinium/formoterol FDC 400/12 μg and FDC 400/6 μg compared with placebo and the monotherapies [23, 24]. Lung function improvements were generally greater with the 400/12 μg dose compared with the 400/6 μg dose [23, 24]. This additive effect of dual bronchodilator use has been reported in several studies with other bronchodilator combinations and is not surprising given that LAMAs and LABAs have different modes of action [15, 16]. However, there is less conclusive evidence that this additional bronchodilation is associated with incremental symptom benefits. The current pooled analysis provides consistent evidence, using various different measurements, that this additional bronchodilation is associated with symptomatic improvements.

In addition to aclidinium/formoterol 400/12 μg, two other LAMA/LABA FDCs (umeclidinium/vilanterol 62.5/25 μg and indacaterol/glycopyrronium 110/50 μg) are available for the treatment of COPD, and several others are in clinical development [31–33]. In a 24-week clinical study, umeclidinium/vilanterol 62.5/25 μg improved TDI focal score versus placebo but not umeclidinium or vilanterol monotherapy at Day 168 [16]. In the SHINE study, indacaterol/glycopyrronium 110/50 μg significantly improved TDI focal score versus tiotropium but not indacaterol or glycopyrronium at Week 26 [15]. It is possible that with a larger dataset, some of the treatment differences between the FDCs and their monotherapy components may have reached statistical significance in these studies. Our pooled analysis has allowed us to examine a larger dataset and extends these results by showing that a dual bronchodilator can provide significantly greater improvements in TDI focal score compared with both its LAMA and LABA monocomponents. Demonstrating the benefit of the combination drug over its monotherapy components for all endpoints is challenging, with most of the comparisons falling short of the MCID for patient-reported outcomes (TDI, SGRQ etc.) [15, 16]. Jones et al. recently highlighted that most of our experience with MCIDs is in the context of comparing an active treatment with placebo, but when adding a second bronchodilator, the additive benefit is likely to be smaller than that between an active treatment and placebo [21]. For example, in the present analysis, the difference in TDI with FDC 400/12 μg versus each of the monotherapies (0.4–0.5 units) was statistically significant but did not reach the MCID. Jones et al. have proposed expressing these endpoints as a ‘minimum worthwhile incremental advantage’; the percentage of patients who would experience improvement at or above the MCID on adding one treatment to another, or comparing two active treatments [21]. In order to assess net benefit, the proportion of patients who deteriorated by the MCID should be reported in addition to the proportion of patients who improved by the MCID, as we have done here. For example, for TDI, approximately 30 % more patients experienced clinically meaningful benefits with the FDC compared with placebo and approximately 8 % more patients experienced clinically meaningful benefits with the FDC compared with either monotherapy.

Dyspnoea is only one of a range of troublesome COPD symptoms that can limit the patient’s ability to perform daily tasks and lead to physical inactivity, social isolation and impaired quality of life [4, 34]. Recently, the importance of night-time and early-morning symptoms of COPD has been recognised. Patient surveys have confirmed that symptoms are at their worst during these times of the day and that these symptoms significantly reduce quality of life [1, 3, 12]. In our pooled analysis, 89 and 94 % of patients experienced night-time and early-morning symptoms at baseline, respectively. This is slightly higher than previous estimates (morning symptoms: 40–81 %; night-time disturbances or symptoms: 58–78 %) in patients with similar airflow limitation (mean % predicted FEV_1_: 52.7–62.3) [3, 11, 12], although it is difficult to draw comparisons given that different questionnaires were employed in each study. What is clear is that symptoms are common amongst patients with COPD at these times of day and our pooled analysis has provided good evidence that aclidinium/formoterol 400/12 μg can improve overall night-time and early-morning symptom severity compared with placebo and both monotherapies.

It should be noted that in the present analysis, the baseline night-time, early-morning and daily (E-RS) symptom scores were relatively low compared with other studies in patients with a similar degree of airflow limitation. Mean baseline overall night-time and early-morning symptom scores ranged from 1.1 to 1.3 units, compared with 1.9 to 2.4 units in the Beier et al. aclidinium monotherapy study (mean % predicted FEV_1_: 55.5–56.0) [35] and mean baseline E-RS total score ranged from 12.2 to 12.9 units compared with 12.5 to 18.2 units in other clinical studies (mean % predicted FEV_1_: 42.2–58.8) [27]. Although statistically significant improvements in these endpoints were observed versus placebo and monotherapy, low baseline scores may have reduced the opportunity to demonstrate benefit in terms of the magnitude of treatment effect.

It is not possible to draw definitive conclusions from comparisons between different studies with LAMA/LABA combinations given the limited data available and differences in study designs and the methods used to assess symptoms. However, in the SHINE study, improvements in the percentage of nights with no awakenings were reported with indacaterol/glycopyrronium 110/50 μg compared with placebo and glycopyrronium, as well as significant increases in the percentage of days with no daytime symptoms compared with placebo and the percentage of days patients were able to perform their usual activities compared with placebo and both monocomponents [15]. Our results extend these findings by demonstrating that aclidinium/formoterol FDC 400/12 μg can provide symptom control over a 24-hour period, improving overall daily, night-time and early-morning symptoms compared with placebo and the monotherapies. Additionally, significant improvements in limitation of early-morning activities were observed versus placebo and both monotherapies. The symptom control achieved over a 24-hour period with aclidinium/formoterol FDC may be a result of its BID administration. Aclidinium monotherapy BID has been shown to improve overall daily, night-time and early-morning symptoms of COPD [35]. Additionally, formoterol BID in combination with an ICS has been shown to improve daily symptoms and reduce night-time awakenings [36, 37]. However, head-to-head studies of once-daily versus twice-daily LAMA/LABA combinations will be required to address this question.

Aclidinium/formoterol FDC 400/12 μg reduced the rate of moderate or severe HCRU exacerbations and EXACT exacerbations of any severity versus placebo. Additionally, the time to first HCRU (moderate or severe or any severity) or EXACT exacerbation (any severity) was significantly reduced with FDC 400/12 μg compared with placebo. Importantly, this benefit was observed in a population not selected for exacerbation history, making the findings relevant to a wider spectrum of patients with COPD. A previous study of a dual bronchodilator in COPD (glycopyrronium [LAMA] and indacaterol [LABA]; the SPARK study) reported significant improvements in exacerbation rates with the combination versus glycopyrronium and tiotropium alone [38]. However, this study benefited from an enriched patient population with more severe airflow limitation (GOLD Stage III and IV) and ≥1 moderate exacerbation in the previous year. Further investigation of aclidinium/formoterol FDC 400/12 μg is required in studies specifically designed to assess exacerbations.

ICS are commonly prescribed in combination with long-acting bronchodilators for the treatment of patients with COPD who are at high risk of exacerbation [39] and it is therefore important to examine the impact of ICS use on treatment outcomes. Stratification of the data by concomitant ICS use demonstrated that FDC 400/12 μg improves TDI by the MCID compared with placebo, regardless of concomitant ICS use. However, significant improvements in TDI with FDC 400/12 μg versus the monotherapy components were only observed in patients who were not using ICS, suggesting that dual bronchodilation provides additional symptom benefit over monotherapy in such patients. HCRU and EXACT exacerbation rates were significantly reduced with FDC 400/12 μg compared with placebo in patients using ICS, but not in those who were not using ICS. However, as expected, exacerbation rates were higher in patients who were prescribed ICS, making it easier to demonstrate a treatment effect in these patients.

## Conclusions

We have shown that a novel, BID FDC of aclidinium and formoterol, administered over six months, significantly improves endpoints that are important to patients with COPD, namely, symptoms (TDI; daily, night-time and early-morning symptoms), relief-medication use and both reported and unreported exacerbations (moderate or severe exacerbations measured by HCRU and exacerbations of any severity measured by EXACT). Improvements in lung function with the FDC versus monotherapy were expected, in line with other recent combination data. However, pooling data from two studies of near-identical design has allowed a more comprehensive investigation of the benefits of the FDC in terms of symptoms and exacerbations. The results are consistent with the improvements in bronchodilation observed in the individual studies and confirm that aclidinium/formoterol FDC 400/12 μg BID may be an effective new treatment option for patients with moderate to severe COPD.

## Additional file

Additional file 1:
**Supplementary information.**

## Abbreviations

AE: Adverse event
BID: Twice daily
CI: Confidence interval
COPD: Chronic obstructive pulmonary disease
EMSCI: Early-Morning Symptoms of COPD Instrument
E-RS: EXAcerbations of Chronic pulmonary disease Tool [EXACT]-Respiratory Symptoms questionnaire
EXACT: EXAcerbations of Chronic pulmonary disease Tool
FDC: fixed-dose combination
FEV_1_: Forced expiratory volume in 1 s
GOLD: Global initiative for chronic Obstructive Lung Disease
HCRU: Healthcare Resource Utilisation
ICS: Inhaled corticosteroids
ITT: Intent-to-treat
LABA: Long-acting ß_2_-agonist
LAMA: Long-acting muscarinic antagonist
LS: Least squares
MCID: Minimum clinically important difference
NiSCI: Night-time Symptoms of COPD Instrument
RR: Rate ratio
SGRQ: St George’s Respiratory Questionnaire
TDI: Transitional Dyspnoea Index

## Footnotes

1 Registered trademark of AstraZeneca group of companies; for use within the USA as Pressair® and as Genuair™ within all other licensed territories.
