# Supplementary material for: Aclidinium bromide and formoterol fumarate as a fixed-dose combination in COPD: pooled analysis of symptoms and exacerbations from two six-month, multicentre, randomised studies (ACLIFORM and AUGMENT)
Source: Respir Res. 2015 Aug 2;16(1):92. doi: 10.1186/s12931-015-0250-2 (PMC4531806; doi:10.1186/s12931-015-0250-2)
Supplement: Additional file 1: — Supplementary information. [file 12931_2015_250_MOESM1_ESM.docx]

**Additional File 1: Online supplement**

**TDI three dimension scores**

FDC 400/12 µg significantly improved TDI three dimension scores (change in functional impairment, change in magnitude of task and change in magnitude of effort) at all time points compared with placebo (all p<0.001) and formoterol (all p<0.05) and at Weeks 12 and 24 compared with aclidinium (all p<0.05).

**TDI stratified by GOLD group**

Sub-analyses of TDI by GOLD group had low statistical power due to the reduction in patient numbers that is a consequence of stratification; patient numbers were particularly low in GOLD groups A and C (number of patients in the ITT population who had sufficient data for GOLD classification and had TDI data available at Week 24: group A: n=252; group B: n=1258; group C: n=89; group D: n=1117). When TDI data were stratified by GOLD group, FDC 400/12 µg significantly improved TDI focal score compared with placebo in GOLD groups A, B and D, but not group C (Figure S1). FDC 400/12 µg also significantly improved TDI focal score compared with aclidinium monotherapy in GOLD groups A and D (Figure S1).

**E-RS domain scores and responders**

Over 24 weeks, FDC 400/12 µg significantly improved scores in the E-RS breathlessness, cough and sputum and chest symptoms domains versus placebo (p<0.001; Figure S2). Additionally, E-RS scores in the breathlessness domain were significantly improved with FDC 400/12 µg versus formoterol (p<0.01) and aclidinium (p=0.001), and E-RS scores in the chest symptoms domain were significantly improved versus aclidinium (p<0.05) but not formoterol (Figure S2). A greater number of patients in the FDC 400/12 µg group achieved the proposed MCIDs in the breathlessness, cough and sputum and chest symptoms domains compared with placebo and monotherapy, and FDC 400/12 µg significantly increased the odds of achieving the MCID in each domain versus placebo (Table S1).

**Individual night-time and early-morning symptoms, limitation of early-morning activities and nocturnal awakenings**

Improvements in individual night-time and early-morning symptoms (cough, wheezing, shortness of breath and difficulty bringing up phlegm) were numerically greater with FDC 400/12 µg compared with each monotherapy, although not all comparisons reached statistical significance (night-time symptoms: p<0.05 vs aclidinium for wheezing and vs both monotherapies for shortness of breath; early-morning symptoms: p<0.05 vs aclidinium for cough and difficulty bringing up phlegm, and vs both monotherapies for wheezing and shortness of breath; no other comparisons reached statistical significance). Improvements in limitation of early-morning activities were significantly greater with FDC 400/12 µg compared with formoterol and aclidinium (both p<0.05) and nocturnal awakenings were significantly improved with FDC 400/12 µg compared with aclidinium (p<0.05) but not formoterol.

**Rate of COPD exacerbations, stratified by GOLD group**

Similar to the TDI sub-analysis, the analysis of exacerbations stratified by GOLD group had low statistical power due to low patient numbers (number of patients in the ITT-exacerbations population who had sufficient data for GOLD classification: group A: n=306; group B: n=1522; group C: n=99; group D: n=1392). When patients were stratified by GOLD group, the rate of HCRU exacerbations was higher in GOLD groups B (0.28–0.38 exacerbations per patient per year) and D (0.53–0.79 exacerbations per patient per year) compared with A (0.17–0.25 exacerbations per patient per year) and C (0.07–0.23 exacerbations per patient per year). A significant reduction in the rate of HCRU exacerbations of any severity was observed with FDC 400/12 µg versus placebo in GOLD group D (Figure S3A). The rate of EXACT exacerbations was similar in GOLD groups A (0.73–1.55), B (1.11–1.67) and D (1.34–1.56), and lower in GOLD group C (0.49–0.89). FDC 400/12 µg significantly reduced the rate of EXACT exacerbations versus placebo in GOLD groups A and B (Figure S3B). Monotherapy data are presented in Figure S3.

**Table S1. E-RS responders**

| **Patients achieving the proposed MCID [1], %** | **FDC 400/12 µg (n=720)** | **Aclidinium 400 µg (n=722)** | **Formoterol 12 µg (n=716)** | **Placebo (n=525)** |
| --- | --- | --- | --- | --- |
| Breathlessness (MCID 1 unit) | 47.0 | 40.4 | 37.9 | 33.2 |
| OR vs placebo | 1.8^***^ | 1.6^**^ | 1.3 |  |
| Cough and sputum (MCID 0.7 units) | 41.6 | 36.0 | 34.0 | 30.7 |
| OR vs placebo | 1.6^**^ | 1.2 | 1.2 |  |
| Chest symptoms (MCID 0.7 units) | 41.9 | 37.7 | 36.1 | 32.6 |
| OR vs placebo | 1.5^**^ | 1.3 | 1.2 |  |

Data are for the pooled ITT-exacerbations population

^***^p<0.001, ^**^p<0.01 vs placebo

COPD, chronic obstructive pulmonary disease; E-RS, EXAcerbations of Chronic pulmonary disease Tool (EXACT)-Respiratory Symptoms questionnaire; FDC, aclidinium/formoterol fixed-dose combination; ITT, intent-to-treat; MCID, minimum clinically important difference; OR, odds ratio; TDI, Transitional Dyspnoea Index

**Figure S1. Improvement in TDI at Week 24, stratified by GOLD group**

**
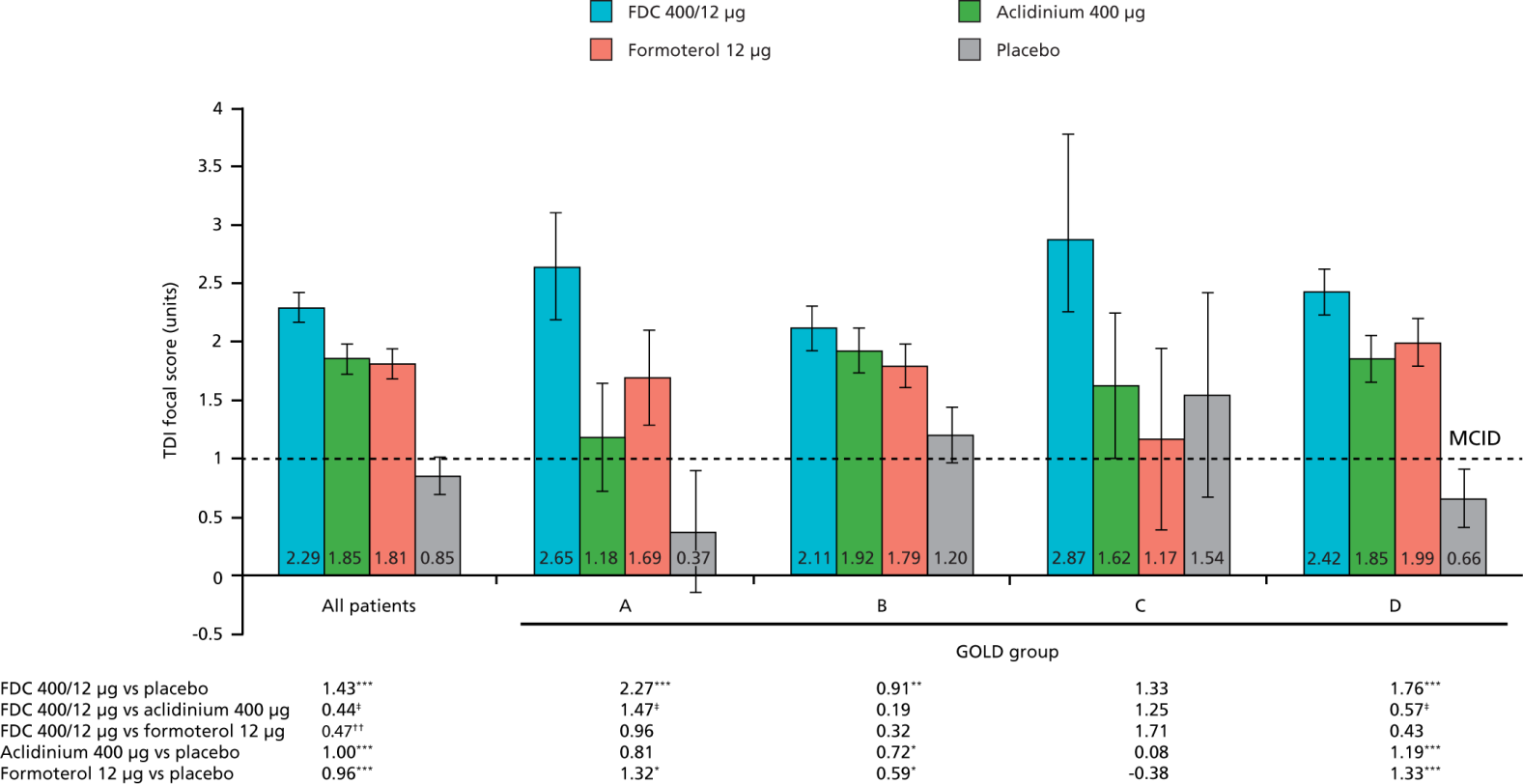
**

Data are LS means ± SE for the pooled ITT population; ^***^p<0.001, ^**^p<0.01, ^*^p<0.05 vs placebo, ^‡^p<0.05 vs aclidinium, ^††^p<0.01 vs formoterol

FDC, aclidinium/formoterol fixed-dose combination; ITT, intent-to-treat; LS, least squares; MCID, minimum clinically important difference; SE, standard error; TDI, Transition Dyspnoea Index

**Figure S2. Change from baseline in E-RS total score and domain scores over 24 weeks**

**
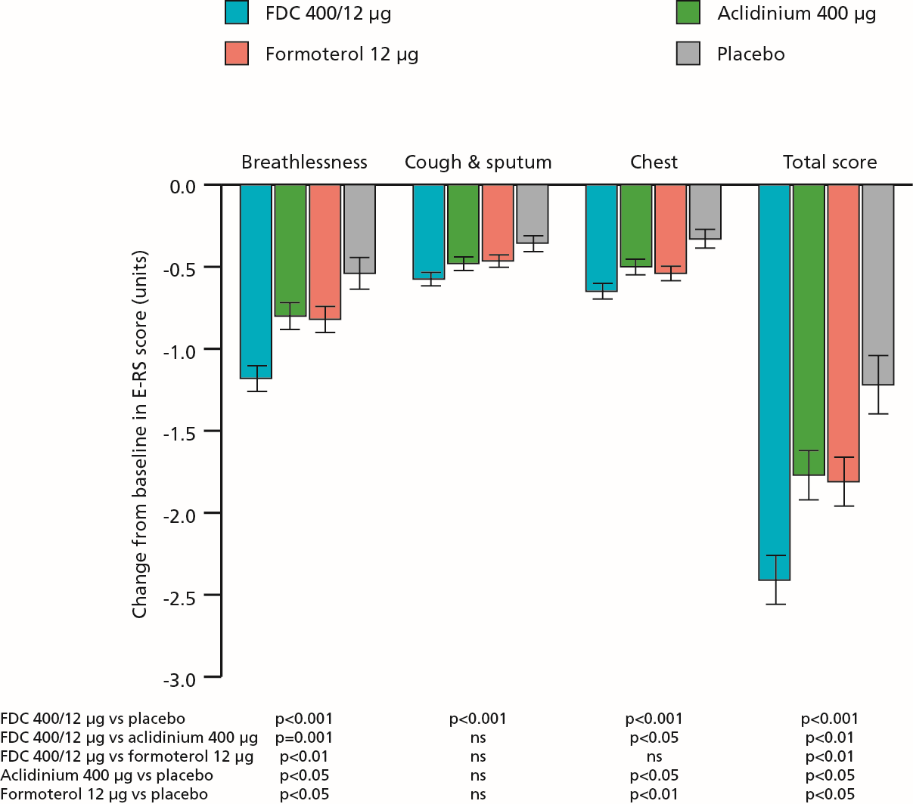
**

Data are LS means ± SE for the pooled ITT population

E-RS, EXAcerbations of Chronic pulmonary disease Tool (EXACT)-Respiratory Symptoms (E-RS) questionnaire; FDC, aclidinium/formoterol fixed-dose combination; ITT, intent-to-treat; LS, least squares; ns, not significant; SE, standard error

**Figure S3. Rate of COPD exacerbations of any severity based on HCRU (A) and EXACT (B) definitions, stratified by GOLD group**

**
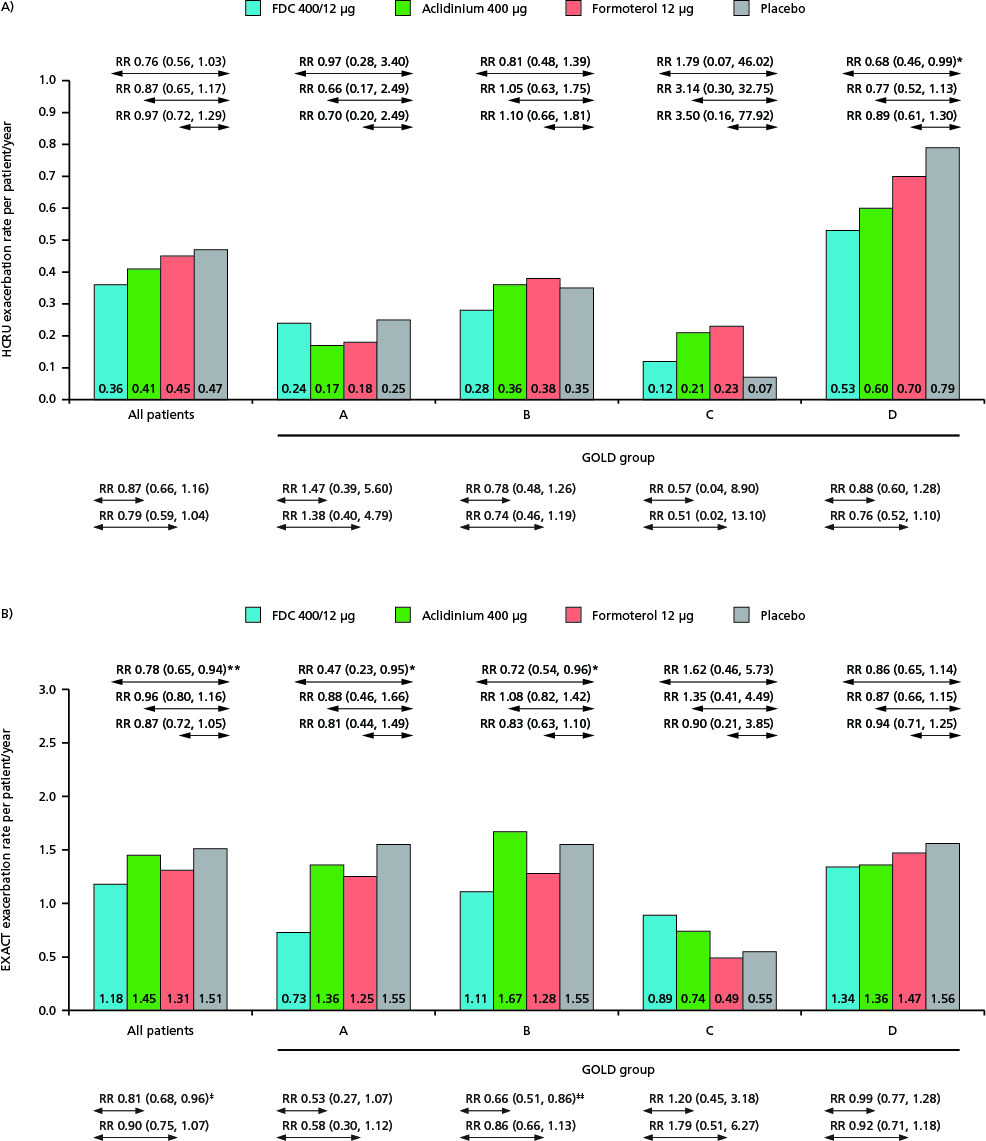
**

Data are LS means and RR (CI) for the pooled ITT exacerbations population; ^*^p<0.05, ^**^p<0.01 vs placebo; ^ǂ^p<0.05 vs aclidinium

CI, confidence interval; COPD, chronic obstructive pulmonary disease; EXACT; EXAcerbations of Chronic pulmonary disease Tool; FDC, aclidinium/formoterol fixed-dose combination; HCRU, Healthcare Resource Utilisation; ITT, intent-to-treat; LS, least squares; RR, rate ratio

**Figure S4. HCRU and EXACT exacerbations, stratified by concomitant ICS use
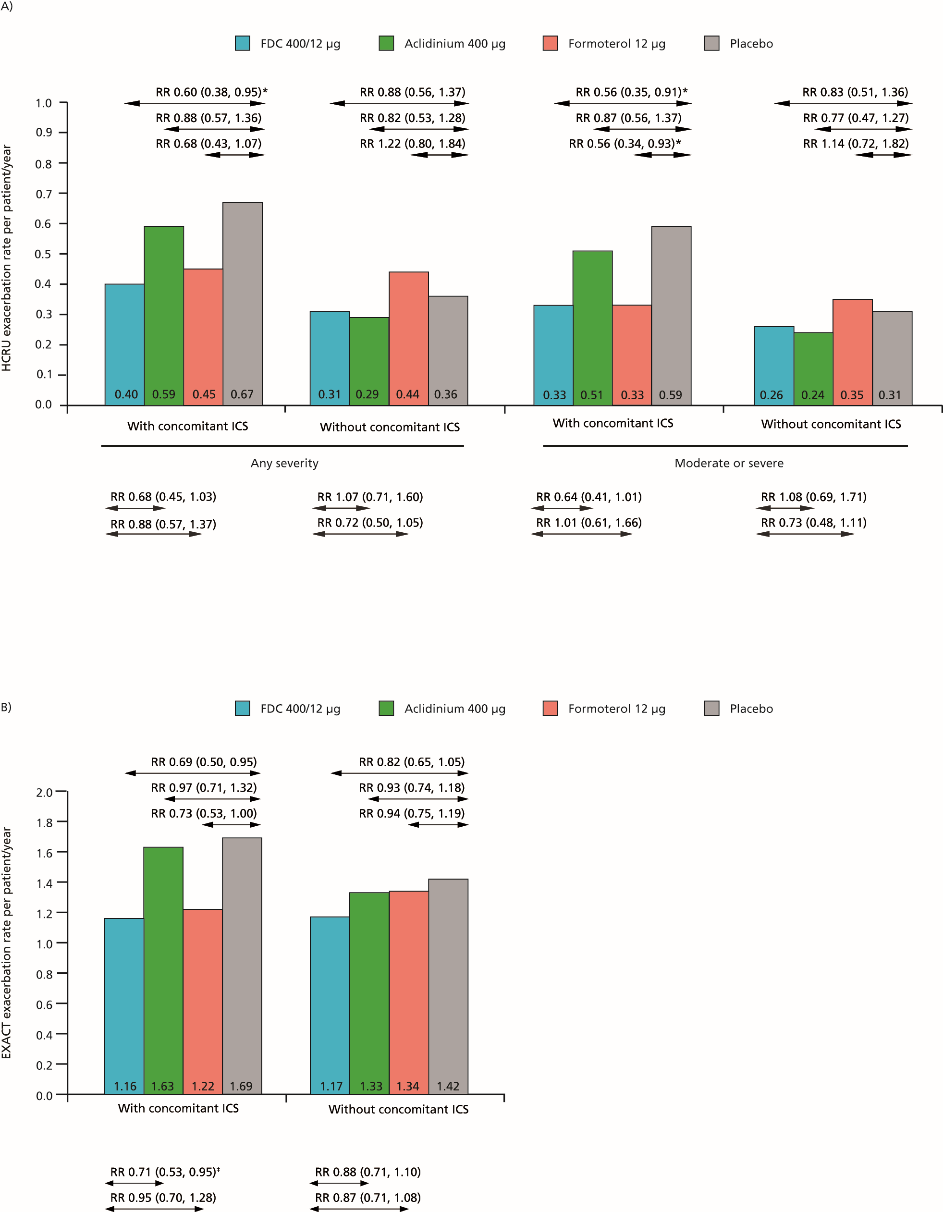
**

Data are LS means and RR (CI) for the pooled ITT exacerbations population; ^*^p<0.05 vs placebo; ^ǂ^p<0.05 vs aclidinium

CI, confidence interval; COPD, chronic obstructive pulmonary disease; EXACT, EXAcerbations of Chronic pulmonary disease Tool; FDC, aclidinium/formoterol fixed-dose combination; HCRU, Healthcare Resource Utilisation; ITT, intent-to-treat; LS, least squares; RR, rate ratio

**References**

1. Leidy NK, Murray LT, Monz BU, Nelsen L, Goldman M, Jones PW et al. Measuring respiratory symptoms of COPD: performance of the EXACT-Respiratory Symptoms Tool (E-RS) in three clinical trials. Respir Res 2014;15:124.
